# Three-Dimensional Bronchovascular Modelling in Sublobar Pulmonary Resection: A Tool for Personalised Thoracic Surgery

**DOI:** 10.3390/jpm16060335

**Published:** 2026-06-22

**Authors:** Victor A. Shahen, Cheng-Hon Yap

**Affiliations:** 1 Department of Cardiothoracic Surgery, St Vincent’s Hospital Melbourne, Fitzroy, VIC 3065, Australia; 2 Department of Cardiothoracic Surgery, University Hospital Geelong, Geelong, VIC 3220, Australia; chenghonyap@gmail.com; 3 Department of Epidemiology and Preventive Medicine, Monash University, Melbourne, VIC 3800, Australia; 4 School of Medicine, Deakin University, Melbourne, VIC 3125, Australia

**Keywords:** 3D modelling, sublobar resection, segmentectomy, surgical planning, lung cancer

## Abstract

Sublobar pulmonary resection has become an increasingly adopted approach for early-stage non-small cell lung cancer, driven by evidence that anatomical segmentectomy can achieve oncological outcomes comparable to lobectomy in selected patients. Safe execution of sublobar resection depends on accurate preoperative identification of segmental bronchovascular anatomy, which demonstrates substantial variability. Conventional two-dimensional (2D) computed tomography (CT) imposes significant limitations on anatomical interpretation, particularly at the segmental and subsegmental level. Three-dimensional (3D) bronchovascular modelling provides patient-specific representations of segmental anatomy and relationships that address these limitations. This narrative review examines the current and emerging roles of 3D modelling in personalised thoracic surgery. It discusses the anatomical basis for its application, the limitations of conventional imaging, and the contribution of 3D modelling to preoperative planning and intraoperative decision making. It also considers broader applications, current limitations, and future directions, with emphasis on how patient-specific 3D modelling can support more tailored operative strategies and more individualised surgical care.

## 1. Introduction

Sublobar pulmonary resection has become increasingly adopted in the management of early-stage non-small cell lung cancer (NSCLC) [1,2]. This reflects a shift toward achieving oncological adequacy with maximal preservation of functional lung parenchyma [1,3]. Its expanding use has been driven by the growing detection of small peripheral lesions through screening programs and incidental imaging [4,5]. Randomised trials have demonstrated that anatomical segmentectomy can achieve oncological outcomes comparable to lobectomy in selected patients, supporting its increasing adoption in thoracic surgery [1,2].

Lobectomy involves resection of an entire pulmonary lobe, including its lobar bronchus, arterial inflow, and venous drainage. Historically, this has been the standard surgical approach for NSCLC [6]. In contrast, segmentectomy constitutes an anatomical sublobar resection that excises one or more bronchopulmonary segments while preserving the remaining parenchyma [7]. Sublobar resection therefore requires precise identification of segmental bronchovasculature and accurate delineation of intersegmental planes [8]. Pulmonary anatomy demonstrates considerable interindividual variability in the branching patterns and spatial relationships of arteries, veins, and bronchi [8,9,10,11,12,13]. These variations may significantly influence both the technical conduct and safety of resection [14]. Failure to recognise patient-specific variants may result in misidentification of segmental structures, vascular injury, or compromised resection margins. As surgical approaches become more conservative, reliance on standardised or “textbook” anatomical configurations becomes inadequate.

Preoperative planning has conventionally relied on two-dimensional (2D) computed tomography (CT), which requires surgeons to mentally reconstruct complex three-dimensional (3D) relationships. This cognitively demanding process depends heavily on spatial interpretation and experience, and may obscure subtle but clinically important variants. These limitations are particularly evident in minimally invasive approaches, where restricted visualisation and lack of tactile feedback further challenge intraoperative orientation [15]. Consequently, critical anatomical detail may only become apparent during dissection, limiting opportunities for pre-emptive planning.

3D bronchovascular modelling has emerged as a technique that enables detailed reconstruction of patient-specific pulmonary anatomy from CT data [16,17]. These models offer accurate, manipulable representations of anatomical structures and their spatial relationships, allowing comprehensive preoperative assessment of segmental anatomy and its variations [18]. Operative strategy can thus be tailored to the true anatomical configuration of the individual patient [19]. As experience with these technologies has grown and evidence supporting their clinical utility has accumulated, 3D reconstruction has become increasingly integrated into segmentectomy planning. Reflecting this evolution, recent European Society of Thoracic Surgeons (ESTS) expert consensus recommendations recognise the importance of 3D reconstruction for preoperative anatomical assessment, evaluation of resection margins, and surgical planning [20].

Consequently, 3D reconstruction is increasingly being incorporated into contemporary thoracic surgical practice rather than being viewed solely as an adjunctive or investigational tool. However, despite the growing clinical adoption, the evidence base remains predominantly observational and is largely derived from retrospective single-centre studies. This review therefore examines the current and emerging roles of 3D modelling in personalised thoracic surgery, focusing on its application in sublobar pulmonary resection.

## 2. Literature Search Approach

This manuscript was prepared as a narrative review. A targeted search was undertaken to identify studies relevant to 3D anatomical modelling, pulmonary anatomical variation, sublobar pulmonary resection, and surgical planning. Relevant articles were identified through searches of PubMed/MEDLINE using combinations of the terms “CT imaging”, “3D modelling”, “3D reconstruction”, “anatomical variation”, “pulmonary anatomy”, “bronchovascular anatomy”, “bronchial anatomy”, “pulmonary arterial anatomy”, “pulmonary venous anatomy”, “lung cancer”, “segmentectomy”, “sublobar pulmonary resection”, “thoracic surgery”, “surgical planning”, and “operative decision-making”. Additional articles were identified through manual review of reference lists from selected publications and citation tracking.

Articles were selected on the basis of their contribution to the current understanding of patient-specific bronchovascular modelling and its application to sublobar pulmonary resection. Priority was given to original clinical studies, anatomical investigations, review articles, and technical reports that informed one or more of the major themes of the review. These themes included anatomical variation, limitations of conventional imaging, 3D reconstruction techniques, operative planning, intraoperative decision making, broader clinical applications, implementation barriers, and future directions in personalised thoracic surgery.

Given the narrative nature of this review, a formal systematic review methodology was not applied. Predefined systematic inclusion and exclusion criteria, PRISMA reporting, quantitative synthesis, and formal risk-of-bias assessment were not performed. The aim was to provide a focused narrative synthesis of representative literature relevant to the role of 3D bronchovascular modelling in personalised thoracic surgery.

## 3. Segmental Pulmonary Anatomy and Anatomical Variability

The pulmonary bronchovascular tree is organised according to bronchopulmonary segments. In the right lung, the upper lobe is divided into apical (S1), posterior (S2), and anterior segments (S3). The middle lobe comprises medial (S4) and lateral (S5) segments, while the lower lobe includes a superior segment and basal segments (S7–S10). In the left lung, the upper lobe consists of the apicoposterior (S1 + S2) and anterior segments (S3), and the lingular segments (S4–S5), while the left lower lobe mirrors the right. Each segment is supplied by a segmental bronchus (B1–B10) accompanied by corresponding arterial (A1–A10) and venous (V1–V10) branches [10,21]. This segmental organisation forms the anatomical basis for sublobar resection. However, bronchovascular anatomy demonstrates substantial variability at both the segmental and subsegmental level, which must be accounted for during surgical planning [8,13].

Pulmonary arterial branching is particularly variable, with differences in the origin, number, and course of segmental vessels well documented in anatomical and radiological studies [10,11,14,21,22,23]. In the right upper lobe, the pulmonary artery typically forms a superior trunk giving rise to the apical (A1) and anterior (A3) segmental arteries, while the posterior ascending artery (A2) often arises separately from the interlobar pulmonary artery. Alternative patterns are common, including a shared trunk (trifurcation), bifurcation with a separate branch, or three independent sequential branches [10,21,23]. In the left upper lobe, the lingular arteries (A4–A5) may arise from a common trunk or as independent branches from the interlobar artery [10,23]. In the lower lobes, variability most commonly involves shared or closely related origins of the superior segmental artery (A6) and the basal arteries (A7–A10), as well as variation in the number and branching pattern of basal vessels [10,14].

Pulmonary venous anatomy shows even greater heterogeneity, which is clinically important due to its role in defining intersegmental planes. Intersegmental veins are classically described as running within the plane between adjacent segments and are widely used as anatomical landmarks during dissection [24]. In practice, venous drainage frequently diverges from this pattern, with anomalous drainage into adjacent segmental or lobar veins commonly observed [8,13]. In the upper lobes, segmental veins typically converge to form the superior pulmonary vein, either as discrete segmental branches or via early confluence into a common trunk. In the lower lobes, the superior segment vein (V6) may drain independently or join the basal venous system (V7–V10), which itself may form a single basal trunk or multiple smaller tributaries [25].

Bronchial anatomy is generally more consistent than vascular anatomy but demonstrates variation in branching patterns. Recognised variants include altered branching order, displaced or anomalous origins, and accessory bronchi [8,9,26,27]. In the right upper lobe, the bronchus most commonly divides into the apical (B1), posterior (B2), and anterior (B3) bronchi in a trifurcation pattern. However, bifurcation patterns and less common variants have also been described [26]. In the lower lobes, the superior segment bronchus (B6) typically arises separately from the basal bronchial trunk (B7–B10), but variation in this relationship is not uncommon [28].

3D CT angiography and bronchography studies confirm that variant configurations are present in a substantial proportion of patients, and many of these variants are clinically relevant [14,17,26]. These findings highlight that “standard” anatomy represents only one of multiple possible configurations, and deviation from textbook descriptions should be anticipated. Recognition of these patterns on preoperative imaging provides the anatomical basis for interpreting patient-specific variation and supports subsequent surgical planning.

## 4. Limitations of Conventional Imaging

Preoperative planning for pulmonary resection is primarily based on CT imaging, which provides high-resolution anatomical detail but remains inherently 2D in its native format. Although multiplanar reconstruction is available, interpretation requires mental integration of axial, coronal, and sagittal images to construct a coherent 3D understanding of bronchovascular relationships. This process is particularly challenging at the segmental and subsegmental level, where structures are small, closely related, and frequently variable [8,13]. As a result, clinically important bronchovascular variations may not be consistently recognised on standard CT imaging [17].

These limitations are particularly relevant in sublobar resection, where accurate identification of segmental anatomy is required [24]. 2D CT imaging does not reliably convey the spatial relationships between arteries, veins, bronchi, and tumour when these structures overlap within a single imaging plane [18,29]. This forces interpretation across multiple sequential slices, increasing the risk of misidentification. Specific anatomical features may be particularly difficult to assess [30,31]. Intersegmental veins can be challenging to trace across consecutive images, and their relationships to adjacent segments may be unclear. Small arterial branches or early bifurcations may also be overlooked, especially when contrast timing is not optimised for pulmonary vasculature. These limitations may lead to incomplete anatomical characterisation prior to surgery and increase the likelihood of unexpected intraoperative findings. Studies evaluating the detection of segmental variants on conventional CT have reported that a substantial proportion of clinically significant variants—estimated at up to 62% in some series—may go unrecognised [30,31].

Interpretation of CT imaging is also operator dependent. The ability to reconstruct 3D anatomy from 2D representations varies between individuals and improves with experience and familiarity with anatomical variation. Although experienced surgeons may develop reliable pattern recognition, this remains a learned and subjective process rather than a standardised method of assessment. Consequently, preoperative interpretation and planning may vary between surgeons.

These limitations are further amplified in minimally invasive approaches, where intraoperative orientation is more dependent on preoperative imaging [15]. Discrepancies between anticipated and actual anatomy may result in disorientation, increased operative time, or modification of the surgical plan [18,32]. Enhanced CT protocols and multiplanar reconstruction provide incremental improvements but do not fully overcome the limitations inherent to 2D representations. Accurate assessment of complex spatial relationships still requires mental reconstruction of bronchovascular anatomy from sequential imaging planes, a process that may be challenging in the presence of anatomical variation [17,18]. These limitations have contributed to the increasing use of 3D reconstruction techniques to improve anatomical interpretation and support more reliable preoperative assessment.

## 5. 3D Bronchovascular Modelling

3D bronchovascular modelling enables reconstruction of patient-specific pulmonary anatomy from CT imaging. It generates 3D representations of segmental arteries, veins, bronchi, and tumour relationships that can be thoroughly assessed in a single view. These models are generated from thin-slice, high-resolution CT datasets that are imported into dedicated segmentation software, where anatomical structures are delineated and segmented [33,34]. Segmentation typically employs a combination of threshold-based techniques to isolate lung parenchyma and vessels, region-growing algorithms to extend segmentations along vascular trees, and manual refinement to correct errors [33,34]. The resulting reconstructions allow rotation, selective isolation of structures, and adjustment of transparency, facilitating direct visualisation of bronchovascular relationships that are difficult to interpret on conventional imaging.

The principal advantage of 3D modelling lies in its ability to accurately depict patient-specific anatomical variation [35,36]. Segmental arteries can be traced from their origin to distal branches, venous drainage can be mapped relative to intersegmental planes, and bronchial branching can be assessed in relation to surrounding vasculature. This enables identification of clinically relevant anatomical configurations that may not be apparent on standard CT [30,31]. Tumour position can also be assessed relative to segmental anatomy, allowing estimation of resection margins and feasibility of segmental resection [33].

Several contemporary studies have reported improvements in anatomical characterisation with 3D modelling, particularly when using automated workflows [32,37]. These approaches have demonstrated high accuracy in the identification and classification of segmental bronchovasculature. In a recent retrospective multicenter evaluation, incorporation of 3D reconstruction into preoperative assessment demonstrated improved anatomical variant identification by 8%, reduced interpretation errors by 41%, and improved procedure selection accuracy by 8% compared with CT-based assessment alone [32].

Beyond visualisation, 3D modelling enables preoperative simulation of the planned resection. Surgeons can define the target segment, anticipate the sequence of vascular and bronchial division, and identify potential technical challenges prior to surgery [35]. The use of 3D models for preoperative simulation has been shown to directly inform and alter operative strategy in sublobar resection [33].

Collectively, 3D bronchovascular modelling provides a detailed, patient-specific representation of pulmonary anatomy that supports more accurate interpretation of imaging findings and informs operative planning. In doing so, it addresses key limitations of conventional 2D imaging and represents an important component of contemporary personalised thoracic surgical planning.

## 6. Identification of High-Risk Anatomical Variants

Identification of high-risk anatomical configurations is an important component of sublobar resection planning, as specific bronchovascular variants may increase technical complexity and influence operative strategy if not recognised preoperatively. While anatomical variability is common, a subset of variants has particular clinical relevance due to their potential to alter the expected sequence of dissection or obscure segmental boundaries. 3D modelling facilitates systematic assessment of patient-specific bronchovascular anatomy and its spatial relationships, which may assist with the identification of these variants [33].

### 6.1. Arterial Variants

High-risk arterial anatomy most commonly relates to variation in segmental branching patterns that alter the expected origin or course of vessels. These include shared arterial trunks supplying multiple segments, atypical origins, and early branching patterns. In the right upper lobe, an additional arterial trunk has been reported to arise separately from the pulmonary artery within the interlobar fissure in up to 17% of cases [10,14,26]. This vessel typically courses posteriorly and may supply portions of the posterior (S2) and/or anterior (S3) segments. Its distal and fissural position can resemble a terminal segmental branch, increasing the risk of misidentification during dissection. Another high-risk pattern is a common arterial trunk between the posterior artery (A2) and the superior segmental artery (A6), present in up to 12% of cases [14,22]. This configuration links upper and lower lobe arterial territories through a single proximal vessel, creating a point of vulnerability during proximal control. In the right middle lobe, a rare but clinically significant variant occurs when the lateral (A4) and medial (A5) segmental arteries share a trunk with the upper or lower lobe segmental branches [14,22]. This shared origin may not be immediately apparent in early dissection, particularly when branches diverge distally. In the left upper lobe, high-risk configurations most commonly involve the relationship between the anterior segmental artery (A3) and the lingular segmental arteries (A4–A5), which arise from a single trunk in up to 19% of cases [14]. This reduces anatomical separation between the upper and lingular divisions of the lobe. In the lower lobes, variation in the relationship between the superior segmental artery (A6) and basal arteries (A7–A10) is also important. These vessels may share a short common trunk or arise in close proximity with overlapping courses [10,23]. Overall, these variants mean that dividing a single artery may affect adjacent segments or even other lobes. Careful identification of arterial branching is therefore essential to preserve functional lung parenchyma.

### 6.2. Venous Variants

Venous anatomy represents a critical source of high-risk variation due to its variability in drainage patterns and its role in defining intersegmental planes. Early venous confluence, where veins from multiple segments merge into a single trunk before entering the superior pulmonary vein, limits the ability to distinguish individual venous territories intraoperatively [14,25,38]. In the right upper lobe, a retrobronchial course of the posterior segment vein (V2) is described in up to 8% of cases [25]. As the vein passes posterior to the bronchus, it may be mistaken for a lower lobe or accessory vessel. In the right middle lobe, anomalous venous drainage may occur, with the middle lobe vein draining directly into the left atrium in 25% of cases or dividing into tributaries to both the superior and inferior pulmonary veins in 9% [14,25]. This alters the expected hilar venous anatomy and may obscure identification of middle lobe drainage. In the left upper lobe, lingular venous drainage (V4–V5) is usually separate from the upper division, but variants include drainage into the anterior segment vein (V3) or into the inferior pulmonary vein [12,39]. These patterns reduce distinction between the lingular and upper division venous outflow. In the lower lobes, variation in the superior segment vein (V6) is particularly relevant. It may drain independently, join the basal venous system, or be associated with accessory branches [14]. When V6 joins the basal veins proximally, the boundary between superior and basal segments may be less distinct. Accessory branches may also course posterior to the intermediate bronchus in up to 20% of cases, placing them at risk during dissection [14].

These patterns highlight that pulmonary venous drainage does not consistently correspond to segmental anatomy. This is also true regarding intersegmental veins, which may deviate from their expected course with possible crossover into neighbouring segmental territories or asymmetric drainage patterns [40]. In these situations, venous anatomy no longer reflects true intersegmental planes. This has contributed to the adoption of intraoperative techniques, such as inflation–deflation methods and indocyanine green (ICG) fluorescence, which define functional rather than anatomical intersegmental planes [41,42].

### 6.3. Bronchial Variants

Bronchial variation is less common than vascular variation but may represent a significant source of intraoperative error when present. This is particularly relevant when it alters the expected branching pattern or position of the target segmental bronchus. In the right upper lobe, the classical pattern is trifurcation into apical (B1), posterior (B2), and anterior (B3) bronchi. However, bifurcation patterns occur in approximately 30% of cases, most commonly forming an apicoposterior bronchus (B1 + B2) with a separate anterior bronchus (B3) [26]. Less common variants include absent or “defective” segmental bronchi, where a segment is supplied by an accessory branch arising from an adjacent bronchus [26]. These configurations alter the expected spatial relationships between bronchial and vascular anatomy. Accessory bronchi represent another important source of variation, including a tracheal bronchus arising proximal to the carina to supply part of the upper lobe. As this branch is not encountered within the hilum, it may be overlooked if not identified preoperatively. In the lower lobes, variation in the origin of the superior segment bronchus (B6) may occur, with close proximity to or a shared origin with the basal bronchial trunk (B7–B10) [9]. This reduces the anatomical distinction between superior and basal segmental bronchi. Overall, bronchial variants primarily increase the risk of incorrect identification of target segmental bronchi.

### 6.4. Tumour-Related Considerations

Tumour-related anatomical relationships are equally important in defining surgical risk. The position of the tumour relative to segmental vessels and bronchi determines both the feasibility of anatomical segmentectomy and the ability to achieve adequate oncological margins [7]. Lesions located near intersegmental planes may require extension of resection to ensure sufficient clearance, while tumours abutting major vessels may necessitate modification of the operative approach or conversion to a larger resection [43]. 3D models enable accurate assessment of tumour position in relation to bronchovascular anatomy, allowing preoperative estimation of resection margins.

In practice, these anatomical and tumour-related factors frequently coexist, creating complex patient-specific scenarios. Failure to recognise high-risk configurations preoperatively may result in bleeding, prolonged operative time, compromised oncological margins, or unnecessary loss of functional parenchyma [19]. These risks are amplified in minimally invasive approaches, where surgeons are more reliant on preoperative anatomical understanding [15]. Accurate characterisation of patient-specific anatomy is therefore essential for safe sublobar resection.

## 7. Personalised Surgical Planning

**Figure 1:**
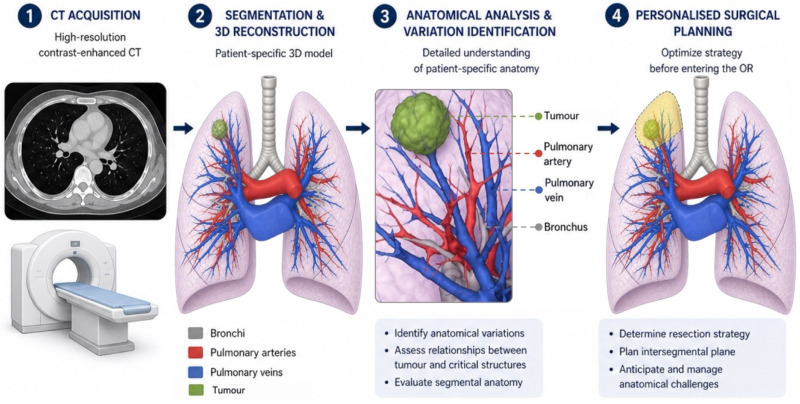
Workflow of three-dimensional bronchovascular modelling in thoracic surgery. High-resolution chest CT imaging is first acquired (1), followed by segmentation of the pulmonary arteries (red), veins (blue), bronchi (grey), and tumour (green) to generate a patient-specific three-dimensional reconstruction (2). The reconstructed model enables detailed anatomical assessment, including identification of bronchovascular variations and evaluation of tumour relationships to surrounding structures (3). These insights support personalised surgical planning, including determination of the extent of resection, anticipated planes, and resection margins, illustrated by the yellow shaded region (4).

3D bronchovascular modelling translates anatomical insight into operative strategy by enabling surgical planning to be tailored to the individual patient. In the context of sublobar resection, this allows operative decisions to be guided by patient-specific anatomy rather than a standard anatomical template. The overall workflow of 3D bronchovascular modelling, from CT acquisition and reconstruction through to use for surgical planning and execution, is illustrated in Figure 1.

### 7.1. Resection Margin Assessment

A central component of the personalised surgical planning process is optimisation of the planned resection. Achieving adequate oncological margins is a fundamental principle of lung cancer surgery, yet this can be challenging in segmentectomy, particularly for lesions located near intersegmental planes, adjacent to major bronchovascular structures, or within anatomically complex segments [7]. 3D visualisation enables precise assessment of tumour position relative to bronchovascular structures and segmental boundaries, allowing evaluation of whether the planned resection is likely to provide sufficient clearance or whether extension of resection may be required [33]. Several studies have demonstrated the utility of 3D reconstruction in this setting. Preoperative 3D modelling has been shown to accurately predict achievement of margins greater than 1 cm, with a reported positive predictive value of 87%, with virtual margins derived from 3D reconstructions demonstrating close agreement with the final pathological margins [44,45]. Additionally, a systematic review and meta-analysis reported that the use of 3D reconstruction and virtual simulation was associated with a significantly lower incidence of inadequate surgical margins during segmentectomy [46]. In a prospective study of 50 patients undergoing segmentectomy planned using patient-specific 3D reconstructions, pathological assessment confirmed radical resection in 98% of cases, although no direct comparison with conventional planning was performed [47]. Collectively, these findings suggest that 3D modelling can improve both preoperative assessment of oncological feasibility and the likelihood of achieving adequate resection margins, consistent with recent ESTS expert consensus recommendations [20].

### 7.2. Complex Segmentectomy Planning

A second important role of 3D modelling is facilitating operative planning in anatomically complex cases. Complexity may arise from anatomical variation or tumour proximity to major hilar structures and intersegmental planes, all of which may coexist. Selection between lobectomy and segmentectomy depends not only on tumour characteristics but also on the feasibility of safely isolating segmental bronchovascular structures within the patient’s anatomy [7,43]. In these situations, detailed understanding of tumour location in relation to surrounding anatomy is essential to determine whether a standard anatomical segmentectomy is technically feasible and oncologically appropriate.

Preoperative 3D assessment allows these relationships to be evaluated before surgery, enabling potential challenges to be identified during the planning phase rather than intraoperatively. This is particularly relevant when lesions are located close to intersegmental planes, where accurate delineation of anticipated planes of division may significantly influence operative planning. While techniques such as inflation–deflation or ICG fluorescence provide valuable intraoperative guidance, they do not contribute to preoperative planning [41,42]. In contrast, 3D modelling enables planned resection boundaries to be assessed before surgery. This may identify situations where extension of resection, combined segmentectomy, or lobectomy is required due to anatomical complexity or tumour proximity to critical bronchovasculature or intersegmental planes.

The clinical significance of this improved preoperative assessment is reflected by a report in which review of patient-specific 3D reconstructions altered the surgical plan in 52% of cases, including changes related to improved tumour localisation in 14% of cases [47]. Consistent with this, improvements in planning performance have also been reported, including an 8% increase in procedure selection accuracy, a 35% reduction in planning errors, and a 25% reduction in planning time [31]. Together, these findings suggest that 3D modelling facilitates more informed determination of the extent of resection and may support consideration of combined segmentectomy or lobectomy where appropriate. This may be particularly valuable in minimally invasive techniques such as video-assisted thoracoscopic surgery (VATS) and robotic-assisted surgery (RATS), where accurate preoperative evaluation is essential for operative planning and execution [15].

### 7.3. Lymph Node Dissection

The influence of 3D modelling on lymph node assessment remains less clear. Current bronchovascular reconstructions primarily focus on pulmonary arteries, veins, bronchi, and tumour anatomy, with no direct visualisation of lymph nodes [32]. While improved understanding of patient-specific hilar anatomy may theoretically assist nodal dissection, a meta-analysis found no significant improvement in lymph node yield with 3D-assisted planning compared with conventional planning. As such, the available evidence does not currently support a meaningful impact of 3D modelling on lymph node dissection.

### 7.4. Local Recurrence Risk

Local recurrence following segmentectomy is closely related to oncological adequacy, particularly achievement of sufficient resection margins and appropriate selection of the extent of resection [1,2]. By facilitating more accurate margin evaluation and operative planning, 3D modelling has the potential to reduce the risk of local recurrence. However, despite the demonstrated benefits, direct evidence demonstrating reductions in local recurrence remains limited. Further prospective studies are therefore required to determine whether the advantages provided by 3D modelling translate into improvements in local recurrence and long-term oncological outcomes.

## 8. Impact on Intraoperative Decision Making

Although 3D bronchovascular modelling is performed preoperatively, much of its practical value is realised in the operating theatre. During sublobar resection, intraoperative decision making occurs within a restricted field of view, where accurate identification of small bronchovascular structures is crucial. Preoperative 3D models of patient-specific anatomy provide a mental reference that helps the surgeon relate what is seen intraoperatively to the expected anatomy.

A major intraoperative benefit is improved orientation. Rather than repeatedly re-establishing spatial position within segmental anatomy, surgeons can proceed with a predefined understanding of the expected order, course, and relationships of the vessels and bronchi [18,32]. This is particularly relevant in thoracoscopic surgery, where exposure is narrow and the view is directional rather than panoramic [15]. In that setting, 3D modelling can reduce the need for exploratory dissection and support more direct progression through key operative steps.

A second important role is intraoperative confirmation. As segmental arteries, veins, and bronchi are exposed, they can be compared against the preoperative model before division [18,33]. This reduces intraoperative uncertainty when the anatomy deviates from expected patterns or when multiple structures are encountered in close proximity. Rather than dictating operative steps, the model serves as a reference against which structures can be validated. This supports more confident decision making at critical points of dissection. This is most relevant in complex segmentectomies, where multiple anatomical variations or deep hilar relationships may complicate intraoperative interpretation. Improved intraoperative interpretation has been associated with measurable procedural benefits [46,48]. However, findings from contemporary individual studies have been variable. A recent randomised controlled trial involving 191 patients found no significant differences in operative time or perioperative outcomes between 3D-assisted and conventional planning [49]. In contrast, a contemporary retrospective study of 265 patients reported reduced intraoperative blood loss, shorter hospital stay, and fewer postoperative complications with 3D-assisted planning [50]. Despite this variability, recent systematic reviews and meta-analyses encompassing 2413 thoracoscopic segmentectomies have reported modest reductions in operative time (up to 13 min) and intraoperative blood loss (up to 16 mL), together with lower rates of conversion and fewer postoperative complications, when 3D modelling was used. Taken together, these findings suggest that 3D-assisted planning may offer perioperative benefits, the magnitude and consistency of which remain uncertain.

Importantly, the potential perioperative benefits associated with 3D modelling should not be interpreted as a replacement for intraoperative judgement. Lung inflation, traction, and distortion may alter the appearance of anatomical structures relative to preoperative imaging. The value of 3D modelling lies instead in providing an accurate anatomical framework against which these differences can be interpreted [19]. In this context, 3D models function as an adjunct to surgical decision making rather than a substitute for operative experience. By facilitating recognition and interpretation of patient-specific anatomy, they may support more controlled and confident execution of segmental resection in anatomically variable settings.

## 9. Applications Beyond Surgical Planning

The utility of 3D bronchovascular modelling extends beyond operative planning and intraoperative guidance, with potential applications in surgical education, patient communication, and multidisciplinary decision making. In these settings, 3D models act as tools for shared interpretation of complex, patient-specific anatomy.

### 9.1. Surgical Education

In surgical education, 3D modelling provides a means of visualising anatomical relationships that are too difficult to appreciate on illustrative diagrams, conventional imaging, or intraoperative exposure. Medical students, junior doctors, and surgical trainees typically rely on 2D representations to mentally reconstruct complex 3D anatomical relationships, a process that is highly dependent on experience and spatial ability. Patient-specific 3D reconstructions allow direct visualisation of segmental arteries, veins, and bronchi in their true spatial configuration, facilitating a more intuitive understanding of anatomical variation [33]. This is particularly valuable in segmentectomy, where detailed knowledge of segmental anatomy is essential but often underdeveloped during early training [7]. Although thoracic-specific evidence is limited, studies in surgical education demonstrate that 3D visualisation improves anatomical understanding and spatial orientation compared with conventional imaging alone across different levels of training [51,52,53]. Exposure to patient-specific 3D models prior to surgery has also been associated with improved procedural comprehension, which may shorten the learning curve of complex procedures [54].

### 9.2. Patient Communication

3D models can enhance patient communication by providing a visual representation of individual anatomy and planned intervention. Explaining thoracic surgical procedures using conventional imaging can be challenging for patients without medical training. 3D reconstruction allows patients to visualise tumour location in relation to surrounding bronchovascular structures and better understand the rationale for the proposed surgical approach. In surgical oncology, use of 3D visualisation has been associated with improved patient understanding and engagement during the consent process [55,56,57,58]. Direct evidence in thoracic surgery is limited, but these findings are plausibly applicable given the inherent complexity of pulmonary anatomy.

### 9.3. Multidisciplinary Planning

Management of lung cancer typically involves collaboration between thoracic surgeons, radiologists, respiratory physicians, and oncologists [59]. While conventional imaging forms the basis of discussion, interpretation of complex anatomical relationships may vary between specialties, and clinically relevant variants may not be identified [30,31]. 3D models provide a patient-specific representation of anatomy that can be viewed collectively, facilitating a more consistent understanding of anatomical relationships. This is particularly relevant when the suitability of sublobar resection is uncertain or anatomical complexity may influence treatment selection. Direct evidence for the impact of 3D modelling in multidisciplinary teams is limited, but available data from surgical planning suggests that 3D visualisation improves communication and agreement between clinicians [18,32,60]. However, these findings require validation in the specific context of multidisciplinary lung cancer discussions.

## 10. Limitations and Barriers to Implementation

Despite increasing interest in 3D bronchovascular modelling, its integration into routine thoracic surgical practice remains constrained by several practical and methodological limitations. These challenges relate primarily to workflow demands, variability in modelling approaches, dependence on imaging quality, and a limited high-level evidence base demonstrating definitive clinical benefit. The principal advantages and limitations of 3D bronchovascular modelling discussed throughout this review are summarized in Table 1.

### 10.1. Workflow Demands and Technical Expertise

A key barrier is the time and expertise required to generate accurate patient-specific models. Segmentation of pulmonary bronchovasculature remains a technically demanding process that requires accurate identification and classification of pulmonary arteries, veins, bronchi, and tumours. Differentiation between arteries and veins, particularly in peripheral branches, often requires careful review of both cross-sectional imaging and 3D reconstructions. The complexity of this process may be further increased by anatomical variation, fused fissures, and suboptimal imaging quality. Consequently, generation of anatomically accurate models depends not only on software capabilities but also on operator familiarity with thoracic anatomy, imaging interpretation, and reconstruction workflows [32].

The learning curve associated with 3D reconstruction represents an additional limitation. Contemporary reconstruction workflows have been developed to improve efficiency and standardisation, with substantial reductions in reconstruction time observed following the initial learning phase. For example, use of a semi-automated 3D Slicer workflow has been reported to reduce model generation times to as little as 20 min following an initial learning curve of approximately five cases [32]. Nevertheless, workflow time and model quality may vary according to operator experience and the software platform utilised. Although studies have demonstrated the feasibility and utility of 3D bronchovascular modelling, there remains limited evidence evaluating the consistency between operators. Consequently, the extent to which segmentation, anatomical classification, and reconstructed anatomy may vary between users remains uncertain. Continued development of automated and AI-assisted segmentation workflows may help reduce operator dependence and improve reproducibility. However, differences between software platforms, segmentation algorithms, and levels of automation may continue to contribute to variability between models.

### 10.2. Software Variability and Lack of Standardisation

Variability between software platforms represents an additional limitation to broader implementation of 3D bronchovascular modelling. A range of commercial and open-source reconstruction platforms are currently available, including Visible Patient (Ethicon, Johnson & Johnson MedTech, Amersfoort, the Netherlands), Synapse Vincent 3D (Fuji Film Co., Ltd., Tokyo, Japan), Materialise Mimics (Materialise NV, Leuven, Belgium), Ziostation 2 (Ziosoft, Inc., Tokyo, Japan), 3D Slicer (Slicer Community, Boston, MA, USA), and OsiriX (Pixmeo SARL, Geneva, Switzerland) [33,35,61,62,63,64,65]. While all can generate clinically useful bronchovascular reconstructions, they differ in their degree of automation, clinical workflow integration, customisability, and cost considerations (Table 2). These differences may influence accessibility and implementation across institutions.

Commercial platforms generally offer more streamlined workflows, greater automation, and dedicated technical support. They are also often designed to integrate directly with institutional imaging infrastructure. However, these advantages are accompanied by substantial financial costs and reduced flexibility for workflow customisation. In contrast, open-source platforms provide greater accessibility, transparency, and adaptability, allowing users to modify workflows and manually refine reconstructions. This flexibility, however, often comes at the expense of increased user input, technical expertise, and workflow complexity.

A related and potentially more significant limitation is the lack of standardisation in reconstruction methodology and reporting. Currently, there is no universally accepted framework for segmentation, anatomical labelling, validation, or quality assessment of bronchovascular reconstructions. Different software platforms utilise distinct segmentation algorithms and reconstruction workflows, while individual users may apply varying thresholding parameters and manual refinement techniques. Consequently, identical imaging datasets may generate different reconstructions depending on the software platform and workflow utilised. The absence of standardisation reduces reproducibility, limits comparability, and complicates interpretation of study findings. Furthermore, direct comparative studies evaluating reconstruction workflow complexity and model accuracy between software platforms remain limited, making objective assessment challenging. Development of consensus reconstruction protocols, reporting standards, and validation frameworks will be important to facilitate broader adoption, improve reproducibility, and enable meaningful comparison of outcomes across institutions.

### 10.3. Imaging Quality Dependence

Model accuracy is also highly dependent on the quality of the underlying CT imaging. High-resolution imaging, appropriate CT protocols, and minimal motion artefacts are required for reliable reconstruction [33]. Suboptimal imaging may impair the delineation of segmental anatomy, particularly when the structures are in close proximity. In these cases, misclassification of vessels may occur, potentially affecting the reliability of models in surgical planning [33].

### 10.4. Evidence Base

The clinical evidence supporting 3D bronchovascular modelling remains limited. Most published studies are retrospective or observational, often conducted in single centres with experienced users. While these studies consistently demonstrate improved anatomical understanding and better operative outcomes, high-quality prospective comparative evidence remains limited [18,35,48]. Consequently, the independent impact of 3D modelling on clinical outcomes remains uncertain.

### 10.5. Cost

Cost considerations represent an additional barrier to adoption. Commercial software platforms and integration into clinical workflows may require substantial financial investment, particularly for smaller centres [66]. Although open-source solutions reduce direct costs, they require greater time investment and technical expertise, representing an indirect resource burden [30,33]. Furthermore, formal cost-effectiveness analyses comparing 3D modelling with conventional imaging, as well as comparisons between different software platforms, remain limited. As such, the balance between implementation costs and potential clinical benefit has yet to be clearly established.

## 11. Future Directions

Although 3D bronchovascular modelling is increasingly incorporated into routine surgical practice, ongoing advances in imaging, automation, immersive visualisation, 3D printing, and data integration are likely to further expand its role beyond anatomical assessment and surgical planning toward broader decision-support applications for personalised thoracic surgery.

### 11.1. Automated Segmentation

Automated segmentation is now available within contemporary reconstruction workflows and represents one of the most rapidly advancing areas of 3D modelling. Nevertheless, bronchovascular 3D modelling often still requires manual review, refinement, and anatomical verification. AI segmentation systems capable of generating bronchovascular models from CT data are already available within several commercial reconstruction platforms and have demonstrated high levels of accuracy, reportedly exceeding 90% for identification and classification of segmental structures [37,67,68]. These technologies facilitate more rapid and scalable generation of 3D models, reducing reconstruction time and improving workflow efficiency. However, differences in segmentation algorithms, workflow integration, and performance across varying imaging quality and anatomical complexity remain important challenges requiring further validation and standardisation.

### 11.2. Intraoperative Navigation

Integration of 3D models into intraoperative navigation represents a second key direction. Rather than serving as static preoperative references, reconstructed models could be linked to real-time guidance platforms to assist with localisation of anatomical structures during dissection. This concept is already established in bronchoscopy, where 3D-guided navigation systems are used to localise target biopsy sites [69]. Application in thoracic surgery would require image-registration techniques capable of aligning preoperative models with the operative field—a significant technical challenge due to intraoperative lung deformation from ventilation and surgical manipulation. Advances in tracking technologies and image processing are likely to improve feasibility.

### 11.3. Extended Reality

Augmented (AR) and virtual reality (VR) platforms allow reconstructed models to be explored or visualised in a more intuitive spatial format compared with conventional imaging. Their use in preoperative planning for thoracic surgery has been associated with modification of operative strategy in over half of cases, improved tumour localisation in 14%, and reduction in resection extent in 10% [47,70]. Intraoperative use in thoracic surgery remains limited, though reality platforms are already established in other surgical fields. In robotic-assisted partial nephrectomy, AR projection of renal vasculature and tumour anatomy guides selective clamping and tumour excision [71,72]. In spinal surgery, AR navigation has been reported to improve the accuracy of pedicle screw placement, with reported success rates approaching 98% [73,74]. These applications support the broader role of immersive technologies in improving operative precision. However, the technical demand of thoracic applications, including variable lung volume, tissue deformation, and instrument handling through a thoracoscopic port, differ substantially from urological and spinal contexts and will require dedicated validation.

### 11.4. 3D Printing

3D printing of patient-specific models from hydrogel-based materials allows production of anatomically accurate replicas. These models possess mechanical properties that approximate human tissue, providing a realistic alternative to cadaveric or animal simulation [75]. They can also be derived from patient-specific imaging to allow for procedural rehearsal in a controlled environment [76]. At present, production cost and time limit routine clinical use. However, improvements in printing speed and workflow integration may allow on-demand generation of patient-specific models for rehearsal of planned resections, particularly in anatomically complex cases. This would represent an evolution from a predominantly educational use toward a practical adjunct in patient-specific surgical planning.

### 11.5. Predictive and Decision-Support Systems

Future approaches are likely to combine anatomical data with clinical, functional, and oncological variables to generate patient-specific risk profiles for surgical complexity, complications, margin adequacy, and postoperative functional preservation. In this setting, 3D pulmonary reconstructions may serve as the anatomical foundation linking structural variation with operative complexity and perioperative outcomes. Postoperative lung function provides one example of how these technologies may be applied. Traditionally, postoperative lung function has been estimated using anatomical segment-counting methods, which assumed that each pulmonary segment contributes equally to the overall pulmonary function. More recently, segment-based high-resolution CT (HRCT) 3D reconstructions have enabled patient-specific quantification of segmental lung volumes and simulation of planned resections [77,78]. Using this approach, postoperative lung function can be predicted based on the volume of preserved lung and anticipated compensatory expansion of the remaining lobes. Early retrospective work has demonstrated closer agreement with observed postoperative pulmonary function than conventional segment-counting methods [77,78].

Similarly, early applications of machine learning methods in oncology have demonstrated potential for predicting patient outcomes [79,80]. Integration of these predictive approaches with patient-specific 3D reconstructions may further enhance surgical planning, risk stratification, and clinical decision making. However, the available evidence remains limited and is derived predominantly from retrospective studies and small cohort analyses.

These developments are interconnected. Automated segmentation enables efficient model generation; intraoperative navigation supports real-time application; immersive visualisation enhances interpretation; 3D printing enables procedural rehearsal; and predictive systems extend this information into decision making. Together, they point toward a model of personalised thoracic surgical care in which planning, execution, and outcome evaluation are aligned around the individual patient’s anatomy and disease characteristics.

## 12. Conclusions

3D bronchovascular modelling represents an important development in thoracic surgery and aligns closely with the broader move toward personalised care. By providing a detailed, patient-specific view of pulmonary anatomy, it addresses a key limitation of conventional 2D imaging—the inability to reliably represent the full extent and significance of bronchovascular variation. This is particularly relevant in sublobar resection, where success depends on accurate identification of segmental anatomy.

Across the stages of care, 3D modelling supports a shift away from standardised approaches toward more individualised decision making. It enables identification of high-risk anatomical variations, informs selection and extent of resection, and supports more accurate delineation of intersegmental planes. Intraoperatively, it provides a validated anatomical reference that supports structure identification as dissection proceeds. Beyond the operating theatre, applications in surgical education, patient communication, and multidisciplinary planning extend its potential impact across the clinical pathway.

Several limitations constrain current implementation. Model generation requires appropriate imaging, time, and technical expertise. Variability between software platforms and the absence of standardised reconstruction and reporting frameworks reduce reproducibility and comparability. Most critically, the evidence base remains predominantly observational. While available studies consistently report clinical utility, high-level prospective evidence confirming an independent impact on operative outcomes and oncological endpoints is lacking.

Advances in automated segmentation, intraoperative navigation, extended reality, 3D printing, and predictive modelling have the potential to address many of these limitations. As these technologies continue to evolve, the current role of 3D modelling in anatomical assessment and surgical planning is likely to expand toward increasingly integrated, data-driven surgical decision making. While 3D bronchovascular modelling is already being incorporated into routine thoracic surgical practice, prospective evaluation will help better define its impact on operative decision making, oncological outcomes, workflow efficiency, and cost-effectiveness. In parallel, standardisation of modelling techniques and reporting frameworks will improve comparability between studies and support continued refinement of its role within contemporary thoracic surgical practice. Ultimately, the ability to tailor perioperative decision making to the unique anatomy of each patient represents a meaningful step toward truly personalised surgical care.

## Figures and Tables

**Table 1:** Advantages and limitations of three-dimensional bronchovascular modelling in thoracic surgery.

Domain	Advantages	Limitations
Anatomical assessment	Improved visualisation of patient-specific bronchovascular anatomy and anatomical variations. Enhanced spatial understanding compared with conventional imaging	Accuracy remains dependent on image quality, software, and operator experience
Resection margin assessment	Facilitates preoperative assessment of tumour location relative to segmental planes and bronchovascular structures. May improve margin adequacy and extent of resection	Margin assessment remains a virtual estimation and must ultimately be confirmed intraoperatively and pathologically
Complex segmentectomy planning	Assists planning of lesions located near intersegmental planes, major hilar structures, or within anatomically complex segments. Helps determine feasibility of a standard anatomical segmentectomy and the appropriate extent of resection	Evidence remains limited and is derived predominantly from retrospective and observational studies
Intraoperative guidance	Provides an anatomical roadmap that may facilitate identification of target structures during surgery	Does not replace intraoperative techniques used to identify intersegmental planes or confirm resection margins
Perioperative outcomes	May reduce operative time, blood loss, conversion rates, and postoperative complications	Current evidence is predominantly retrospective and observational
Clinical implementation	May support minimally invasive and robotic-assisted thoracic surgery workflows	Adoption may be limited by software availability, technical expertise, institutional resources, and lack of standardised reconstruction protocols

**Table 2:** Comparison of commonly used software platforms for three-dimensional bronchovascular reconstruction and surgical planning.

Platform	Category	Automation Level	Customisability	PACS Integration
3D Slicer	Open source	Low	High	No
OsiriX	Open source	Moderate	Moderate	No
Mimics (Materialise)	Commercial	Moderate	Moderate	No
Synapse Vincent 3D (Fujifilm)	Commercial	High	Low	Yes
Ziostation 2 (Ziosoft)	Commercial	High	Low	Yes
Visible Patient	Commercial	High	Low	No

Abbreviations: PACS, Picture Archiving and Communication System.

## Data Availability

No new data were created or analysed in this study.
